# Integration of Conventional Sensors and Laser Doppler Vibrometry for Structural Modal Analysis: An Innovative Approach

**DOI:** 10.3390/s26020418

**Published:** 2026-01-08

**Authors:** Eva Martínez López, Natalia García-Fernández, F. Pelayo, Marta García Diéguez, Manuel Aenlle

**Affiliations:** Department of Construction and Manufacturing Engineering, University of Oviedo, 33203 Gijón, Spainfernandezpelayo@uniovi.es (F.P.); garciadmarta@uniovi.es (M.G.D.)

**Keywords:** Laser Doppler Vibrometry, OMA, structural health monitoring (SHM), non-contact measurements

## Abstract

This study aims to demonstrate the feasibility of a hybrid measurement system that combines Laser Doppler Vibrometry (LDV) and conventional accelerometers for operational modal analysis (OMA) of civil engineering structures. The proposed approach addresses the limitations of traditional accelerometer-based systems, particularly for large-scale or inaccessible structures, by integrating non-contact LDV measurements with conventional sensor data. Experimental tests were conducted on a cantilever beam and a pedestrian laboratory footbridge to validate the hybrid system. The LDV was used to measure velocity at key points, while accelerometers provided complementary reference acceleration measurements. Reflective targets were employed to facilitate non-contact data collection, allowing for the subsequent reuse of these targets for repeated measurements. The velocity data from the LDV were differentiated to obtain acceleration and integrated to estimate displacement, enabling a direct combination with accelerometer data. ARTeMIS Modal software was utilized to process and analyze the collected data, successfully identifying the natural frequencies and vibration modes of both structures. The results demonstrate that the LDV–accelerometer hybrid system effectively captures the dynamic behavior of structures, offering a comprehensive solution for modal analysis without extensive sensor deployment. This approach provides significant advantages in scenarios where traditional methods are impractical, positioning the hybrid system as a promising tool for dynamic analysis and infrastructure monitoring of complex structures.

## 1. Introduction

In recent years, Structural Health Monitoring (SHM) has gained significant importance in civil engineering due to the aging of infrastructures and the increasing demand for their reliability. Structures such as bridges, buildings, and footbridges are constantly exposed to dynamic loads—traffic, wind, and environmental changes—which can weaken their integrity over time. By SHM, engineers can detect early signs of damage, enable timely interventions and prevent catastrophic failures. Therefore, SHM plays a critical role in maintaining the safety and longevity of essential infrastructure [1,2].

Among the various techniques used in SHM, modal-based methods —particularly those based on operational modal analysis (OMA)—have gained prominence in civil engineering [3]. In OMA, the structure is monitored under its normal operating conditions, and the excitation is treated as broadband stochastic (e.g., traffic, wind, pedestrian flows), so that the modal parameters are identified from output-only measurements. In contrast, experimental modal analysis (EMA) relies on input–output data, which is difficult to implement on large in-service structures. Modal-based techniques provide valuable insights into a structure’s dynamic behaviour by identifying modal parameters like natural frequencies, vibration modes, and damping ratios. While this work focuses on operational modal analysis and consistent modal-parameter estimation, it is worth noting that the same measured vibration responses (acceleration/strain/displacement) are also routinely post-processed for damage identification via feature extraction [4,5]. Traditionally, accelerometers have been used for these measurements due to their ability to accurately track vibrations at specific points on the structure. However, accelerometer-based systems require direct contact with the structure which can complicate sensor placement, particularly in large-scale or hard-to-reach infrastructures and may pose safety risks during installation. Moreover, accelerometers often have shorter lifespans than structures they monitor, necessitating frequent maintenance. These limitations have sparked interest in alternative non-contact measurement technologies [6,7,8,9,10].

One of the most promising alternatives is Laser Doppler Vibrometry (LDV), which allows for non-contact vibration measurements, overcoming many of the challenges associated with accelerometers. LDV can capture vibration data from remote or inaccessible areas without requiring physical contact, making it especially useful for monitoring large infrastructures like bridges and dams. Despite these advantages, applying LDV on complex structures such as curved or distant surfaces can be challenging. To address this, recent innovations such as drone-based LDV systems equipped with reflective mirrors have been proposed, extending the reach of LDV and enhancing its flexibility in difficult environments [11,12].

From a sensing perspective, modern LDV systems offer a very wide measurement bandwidth, typically from near (Digital Current) DC up to tens of kilohertz or even megahertz, together with very low noise levels in the velocity signal and vibration amplitudes well below the micrometre level. This makes LDV, in principle, well suited for civil applications, where the relevant modal content usually lies below a few tens of hertz [13,14,15,16,17]. However, the usable performance of LDV on real structures is often governed by practical issues such as the optical effects like speckle and low surface reflectivity [18,19,20,21]. As a result, the effective signal-to-noise ratio depends not only on the intrinsic sensitivity of the vibrometer, but also on stand-off distance, surface condition and environmental conditions in the field. These aspects must be considered when using LDV as a primary response sensor for OMA in civil engineering [22].

In parallel to LDV, several image-based, non-contact measurement techniques such as Digital Image Correlation (DIC) and video-based motion tracking have also been proposed for civil engineering applications [23,24,25,26]. These methods can provide multi-point displacement measurements, using standard or high-speed cameras. However, their performance strongly depends on favourable line-of-sight and lighting conditions, sufficient surface texture or speckle patterns, careful camera calibration, and a trade-off between field of view, spatial resolution and frame rate [27,28,29,30]. In contrast, LDV offers very high sensitivity to small vibration amplitudes, a wide measurement bandwidth and the ability to operate at relatively long stand-off distances with a simple one-dimensional velocity signal and reduced data volume.

The present study introduces an innovative approach by combining LDV with conventional accelerometers for operational modal analysis. The novelty of the work lies in the proposed hybrid measurement framework and data fusion strategy, in which a single reference accelerometer and sequential LDV measurements are combined to obtain consistently scaled modal parameters. While both LDV and accelerometers have been used individually in structural dynamics, the integration of these two systems to derive consistent modal parameters is unexplored. This hybrid method addresses challenges in accessing certain parts of structures and offers a non-contact alternative that simplifies setup, making it particularly suitable for complex or large-scale infrastructures. This hybrid approach takes advantage of the complementary strengths of both sensor types. Accelerometers excel at providing high-resolution local data [31,32], while LDV captures the global response of the structure without the need for extensive sensor deployment [15,33]. Together, they enhance the accuracy of modal analysis and expand diagnostic capabilities, offering a flexible solution for infrastructure monitoring beyond traditional SHM systems. Furthermore, improving these analysis techniques aligns with the goal of prolonging the operational lifespan of critical infrastructures, contributing to the long-term resilience of built environments [2,34,35,36].

This paper is organized as follows. Section 2 describes the proposed measurement methodology and the experimental program, along with the main results, which are discussed in Section 3. Finally, the main conclusions are presented in Section 4.

## 2. Methodology and Experimental Program

This section describes the experimental program developed to validate the proposed hybrid measurement system, which integrates Laser Doppler Vibrometry (LDV) with conventional accelerometers for OMA. The structures analysed in this study included a flat acrylic beam and a laboratory pedestrian footbridge (UniOvi FootBridge [37,38]).

The LDV–accelerometer system consists of using a single reference accelerometer, permanently installed on the structure, while the LDV is sequentially directed to different points of interest to acquire velocity data. In this way, the information from both sensors can be combined to extract consistent modal parameters.

The experimental tests were carried out using a Polytec GHmb (Waldbronn, Germany) Laser Doppler Vibrometer, formed by the sensor head OFV-505 and the OFV-5000 vibrometer controller, which includes an internal velocity decoder module and a displacement decoder module. The OFV-505 sensor head integrates the interferometer, laser and imaging optics in a compact housing, and is equipped with a visible He–Ne laser (λ = 633 nm, Class 2, <1 mW) and auto/remote focus functions, which enable precise alignment and stable measurements at stand-off distances of up to several tens of metres. The modular OFV-5000 controller accepts up to four decoder modules and can cover frequencies from near 0 Hz up to several MHz and velocities ranging from very low ambient-vibration levels to about ±10 m/s, depending on the selected range.

In addition to the LDV, uniaxial piezoelectric accelerometers from PCB Piezotronics Inc. (Depew, NY, USA) were employed, with sensitivities of 100 mV/g and 1 V/g. To validate the signal acquired by the vibrometer, a laser displacement sensor from Acuity (Atlanta, GA, USA) was also used. Furthermore, to ensure control over the initial excitation parameters, an electromechanical shaker capable of generating controlled frequencies was used.

A data acquisition system (DAQ) from National Instruments NI (Austin, TX, USA) was used, incorporating a sound and vibration input module, as well as a voltage input module, both from NI. Signals were processed following standard digital-signal-processing steps, including detrending, band-pass/anti-alias filtering and numerical differentiation/integration when required [39]. Down-sampling (decimation) was applied after anti-alias filtering to focus the analysis on the low-frequency range of interest. All routines were implemented in LabVIEW 2012 for convenience, but the workflow is software-agnostic and can be reproduced in environments such as MATLAB R2020b or Python 3.8.20.

Modal parameters were then identified via Frequency Domain Decomposition (FDD) based on the Singular Value Decomposition (SVD) of the response spectral density matrix (see also Brincker & Ventura [40]).

### 2.1. Preliminary Validation of the Methodology

The objective of this preliminary test is to demonstrate that the data from the two sensors (LDV and accelerometers)—measuring velocity and acceleration, respectively—could be effectively combined.

**Figure 1:**
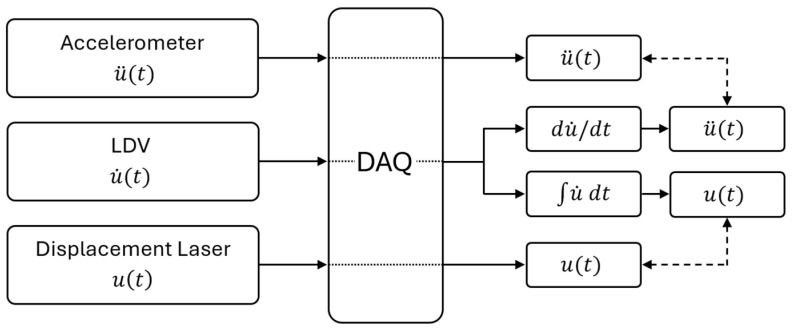
Comparison scheme of displacement, velocity, and acceleration measurements from the LDV, accelerometers, and displacement laser.

The validation method relies on the intrinsic relationships among displacement, velocity, and acceleration. Since the Laser Doppler Vibrometer (LDV) provides velocity as direct output, the signal can be differentiated to yield acceleration, which is directly comparable with the measurements from the accelerometers (Figure 1). Conversely, the same velocity data can be integrated to obtain displacement, enabling comparison with the readings of a laser displacement sensor (Figure 1).

In the proposed workflow, accelerometer and LDV signals are not averaged. Accelerometers are used as simultaneously recorded reference channels, whereas the LDV provides an additional response channel. In this study, the subsequent processing and comparisons are performed in the acceleration domain, where the accelerometer measurements are always taken as the reference; therefore, the LDV velocity time history is numerically differentiated when an acceleration-equivalent signal is required. Nevertheless, a displacement-based workflow is also possible in principle, by using a laser displacement sensor and/or by numerically integrating the LDV velocity signal.

**Figure 2:**
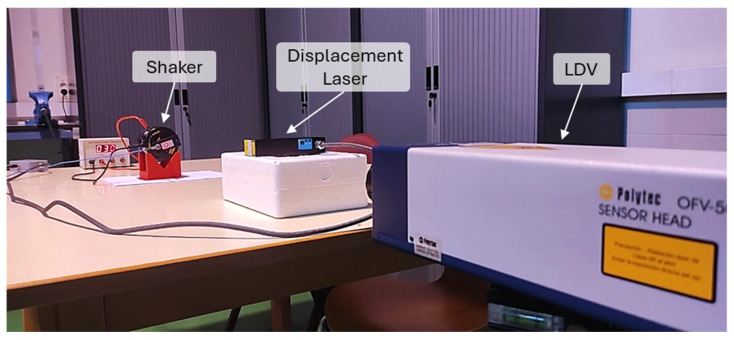
Setup to validate the methodology.

To verify this approach under controlled conditions, an electromechanical shaker was employed. A reflective target was placed on the shaker, and three types of sensors were simultaneously used: the LDV, the accelerometer, and the displacement laser (Figure 2). The shaker introduced controlled vibrations, while the LDV velocity signal was processed through differentiation and integration to obtain acceleration and displacement estimates. These transformed signals were compared with the acceleration obtained from the accelerometer and with the displacement recorded by the laser sensor, showing close agreement in both cases.

**Figure 3:**
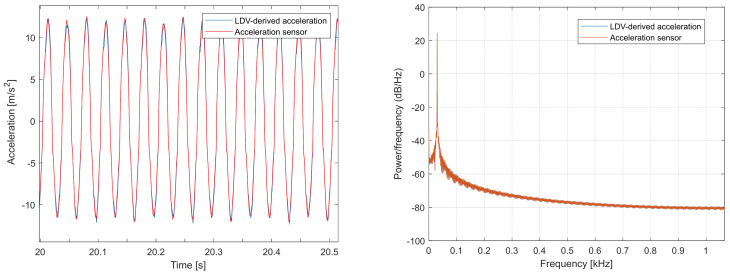
Comparison of acceleration signals obtained from the accelerometer and from the differentiated LDV velocity data for the 30 Hz shaker motion. Baseline: accelerometer sensor.

Figure 3 shows the acceleration-signal comparison for the 30 Hz sinusoidal motion of the electromechanical shaker. The two signals are practically identical (concordance correlation coefficient, CCC = 0.998; Pearson correlation coefficient, r = 0.998; normalized root-mean-square error, NRMSE = 0.019, ≈1.9% of the range).

Although the obtained results were expected given the inherent relationship among displacement, velocity, and acceleration through integration and differentiation, they provide an experimental confirmation of this consistency. These comparisons validate the proposed methodology and demonstrate that LDV measurements can be effectively combined with conventional sensors, ensuring full compatibility across the displacement, velocity, and acceleration domains.

#### Angled Measurements

Uniaxial LDVs present an inherent limitation due to the system itself: they can only detect motion occurring along the direction parallel to the emitted laser beam—that is, out-of-plane motion. This would be the ideal set-up. Moreover, this implies that when the target motion takes place in a plane perpendicular to the beam, the measurements obtained are either inaccurate or non-existent.

**Figure 4:**
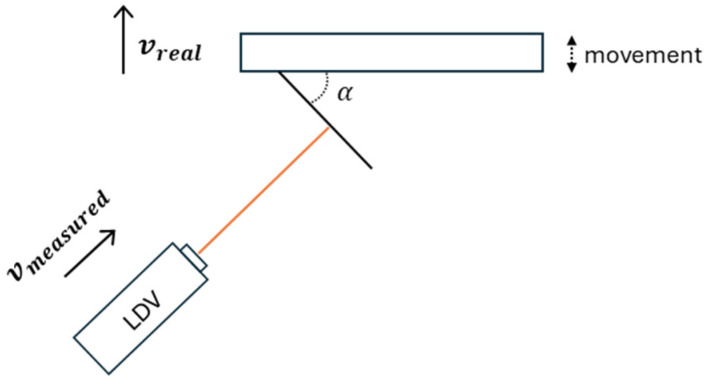
Inclined-target setup for in-plane motion measurement.

To overcome this limitation and enable in-plane motion measurement (non-ideal set-up), inclined targets with a known angle (α) to respect the real measurement movement can be used (Figure 4). The LDV was directed at these targets, effectively projecting the in-plane motion onto an out-of-plane component, which the LDV could then detect [41].

By knowing the inclination angle of the target (α), the actual in-plane motion was obtained using a trigonometric relationship, according to Equation (1).

(1) $$v_{real}=\frac{v_{measured}}{\cos\left(\alpha \right)}$$

**Figure 5:**
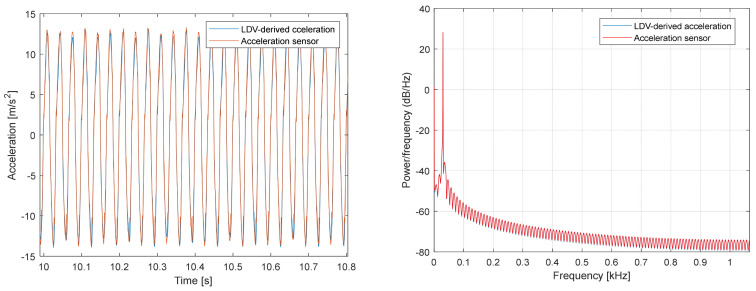
Inclined-target measurements from both accelerometer and LDV-derived signals. Baseline: accelerometer sensor.

These observations were validated using accelerometers, which permitted direct comparison with the acceleration values derived from LDV velocity data. A comparison for an inclined target with α=60° is presented in Figure 5. From the comparison of the two signals; it can be observed that they are practically identical (CCC = 0.995; r = 0.997; NRMSE = 0.032, ≈3.24% of the range).

While Figure 4 shows the ideal setup for an inclined target, the target’s projected motion can also be measured when the laser head is not perfectly normal to the surface. This increases flexibility but also the noise in the received signal.

After these validations, as previously commented, the proposed methodology is applied to two different structures: a cantilever beam and a pedestrian footbridge.

### 2.2. Cantilever Beam Tests

**Figure 6:**
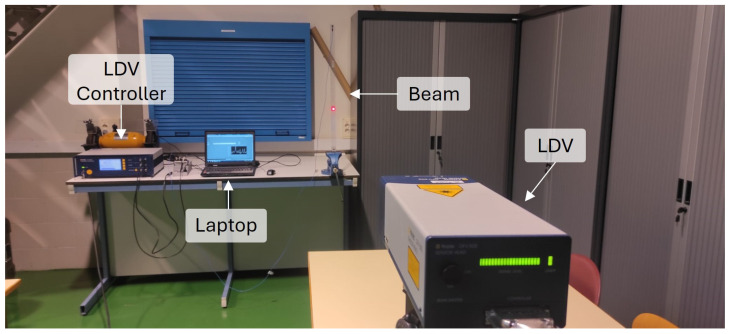
Cantilever acrylic beam.

The first OMA test was performed on a cantilever acrylic beam, 847 mm long with a rectangular cross-section of 27.2 mm × 7.5 mm (Figure 6). Random excitation was manually applied in order to evaluate the structure’s dynamic behaviour and to identify its modal parameters [40].

**Figure 7:**
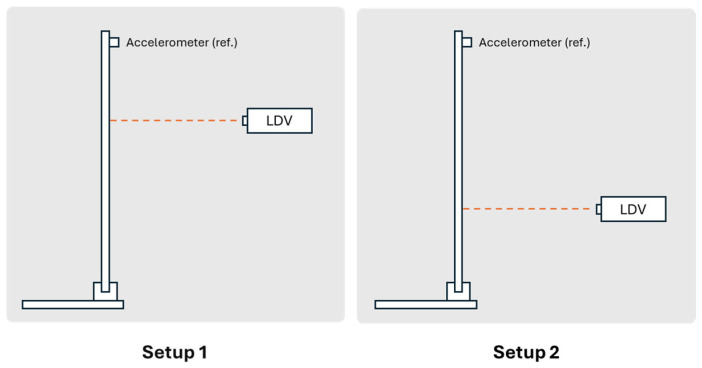
Setup scheme of the cantilever acrylic beam.

The goal of the test was to identify the first three vibration modes of the beam. To achieve this, three equidistant measurement points were selected along the beam and two separate data sets were collected to cover all measurement points. A fixed accelerometer was placed at the free end of the cantilever as a reference point in both data sets, while the other two points were measured independently using the LDV, each in a separate data set (Figure 7).

**Figure 8:**
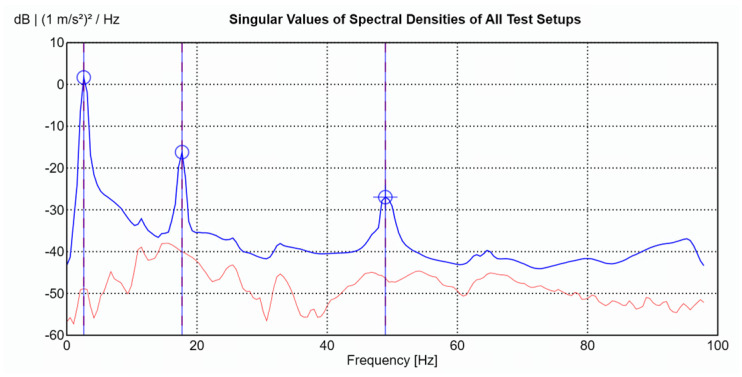
Singular Value Decomposition plot for the cantilever beam modal identification.

For each data set, the LDV was positioned perpendicular to the surface of the beam to measure velocity at the designated point. After measurement, the velocity signals from the LDV were both differentiated to obtain the accelerations at the corresponding points to work with all the signals in the acceleration domain. After acquiring both data sets, the results were combined in ARTeMIS Modal software (Version 8.0.1.7) to identify the natural frequencies and mode shapes of the beam using the Frequency Domain Decomposition (FDD) technique. To improve the low-frequency analysis—where the main structural modes are located—all response signals were decimated to a sampling frequency of 100 Hz before performing the modal identification. The singular value decomposition (SVD) for the FDD identification is presented in Figure 8.

**Figure 9:**
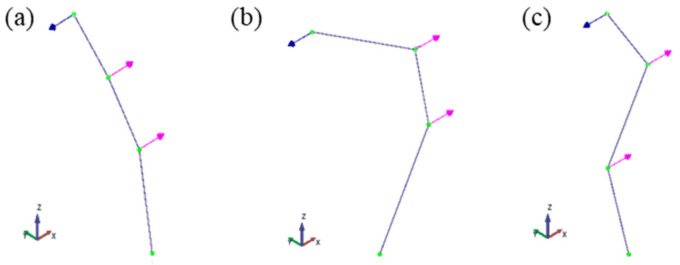
(**a**–**c**) Mode shapes obtained with the proposed hybrid system (accelerometer + LDV).

The extracted natural frequencies and mode shapes are summarized in Table 1 and illustrated in Figure 9, respectively. The natural frequencies are estimated with an error below 1.4%, which can be attributed to the estimation procedures.

The small errors obtained in both natural frequencies and the identified mode shapes demonstrate that the adopted measurement and identification methodology (combining LDV and accelerometers) can be reliably used to determine the modal parameters of the structure under investigation.

### 2.3. Pedestrian FootBridge Test

The last experimental test involved a model of a laboratory pedestrian footbridge, which represents a scenario highly like a real civil structure, thereby increasing the complexity and realism of the study.

**Figure 10:**
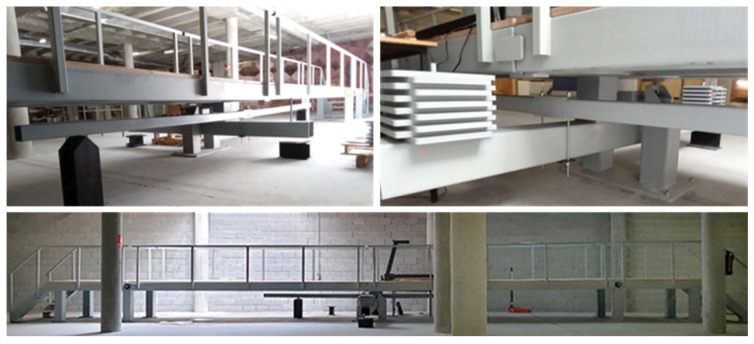
The UniOvi FootBridge.

The test was conducted on the UniOvi FootBridge (Figure 10) located at the University of Oviedo, a full-scale laboratory structure designed to study vertical human-structure interaction [37,38]. The footbridge is 12 m long, 1.5 m wide and 1 m high, and consists of two main steel deck segments connected at midspan by sliding bearings. An adjustment system connected to the centre of the deck allows tuning of both mass and stiffness by modifying the configuration of a seesaw and a flexible beam, thereby enabling the natural frequency of the first mode to be varied between 1.6 and 10 Hz.

**Figure 11:**
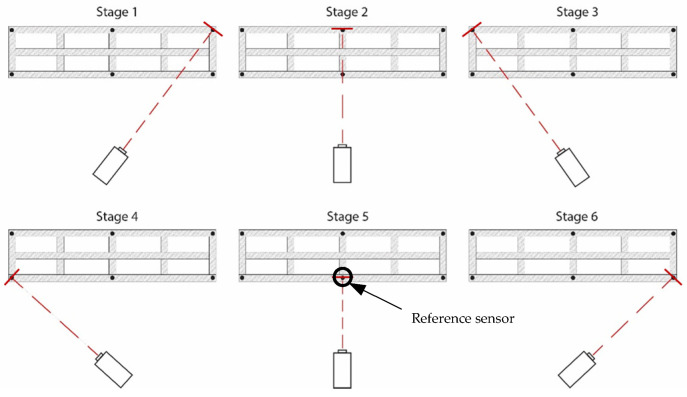
Experimental setup scheme: measurement points and LDV position (top view of the left half of the footbridge deck).

**Figure 12:**
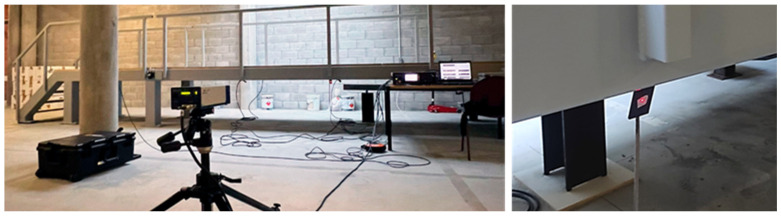
Experimental setup. LDV location (**left**) and targets (**right**).

In this case, the LDV was fixed at a single location and sequentially directed to six inclined targets placed along the footbridge (Figure 11). As the pedestrian structure is at lab location, the height between the floor and the deck is about 1 m. Therefore, it is not possible to use an ideal inclined configuration (Figure 4) where the laser beam will be normal to the target surface. In this case, 70° degrees targets were used, and the laser beam is almost normal to the direction to measure (vertical footbridge motion) (Figure 12). As this can be considered as a limit measurement situation, to improve the received laser measurement, reflective adhesive tape was used in the targets. This configuration reflects a scenario in which the vibration modes of interest are vertical, as typically observed in real bridges but the LDV cannot be operated directly aligned with the direction of movement, e.g., a bridge crossing a river, not accessible structure, etc.

Six accelerometers (1 V/g) were permanently installed along the beam measuring in the vertical direction (black dots in Figure 11), while the LDV was sequentially directed to the same six points to acquire velocity data. That is, each record therefore contains the same reference accelerometer channels plus one LDV channel (sequentially moved), and the LDV contribution is incorporated by using the reference accelerometer rather than by averaging the two systems In total, six data sets were necessary to measure the six points in the structure using the acceleration sensor at stage 5 as reference (see Figure 11). Two methods were then compared: (i) the conventional OMA approach, based solely on the simultaneous measurements from the six accelerometers; (ii) the proposed methodology, which combines the LDV measurements at the six points with a single reference accelerometer, therefore using the 6 data sets. By contrasting these two approaches, the study evaluated the capability of the hybrid LDV–accelerometer system to accurately capture the modal properties of the structure. It is worth noting that, in the proposed hybrid methodology, the LDV velocity signals are differentiated to obtain accelerations that can be combined with the reference accelerometer as well as corrected using Equation (1).

**Figure 13:**
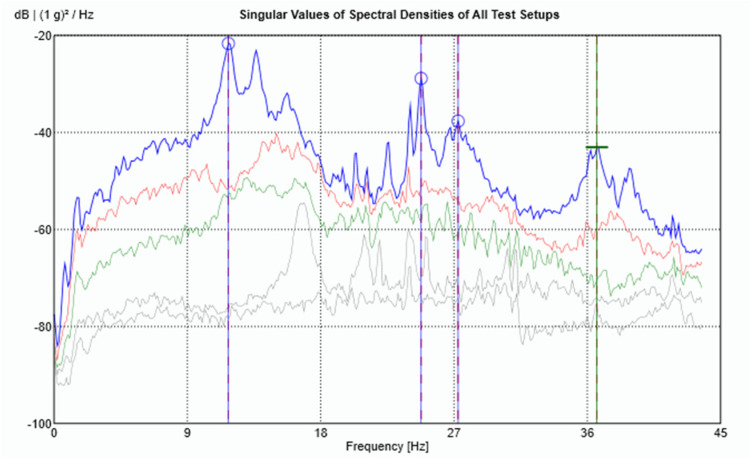
Singular Value Decomposition for the modal parameters identification of the UniOvi FootBridge.

Each measurement lasted approximately five minutes, with excitation provided by one or two people walking on the structure. The data were registers using a sampling frequency of 2132 Hz, which corresponds to the minimum sampling rate allowed by the NI CompactDAQ acquisition system. The signals from both systems were then analysed using ARTEMIS Modal software to identify the modal parameters of the structure. To improve the low-frequency analysis—where the main structural modes are located—all response signals were decimated to a sampling frequency of 50 Hz before performing the modal identification. The natural frequencies obtained with both systems (only accelerometers vs. LDV + one accelerometer) are shown in Table 2. The singular value decomposition (SVD) for the FDD identification is presented in Figure 13. The minor differences in the frequencies, with errors of less than 1.4%, demonstrate the effectiveness of the proposed methodology.

The Modal Assurance Criterion (MAC) between the experimental mode shapes obtained from both systems is presented in Table 3 and indicates reasonable agreement. Note that, because the laser operated in a non-ideal configuration for inclined targets (Figure 4), the signal-to-noise ratio (SNR) is reduced; consequently, mode-shape identification is affected, and the MAC values are lower than expected. Even so, under these limiting conditions—i.e., with the laser beam nearly orthogonal to the measured motion—reasonably good results are still obtained.

Finally, the experimental mode shapes are graphically compared with the numerical mode shapes obtained by M. Dieguez et al. [38] in Table 4. It should be noted that the experimental results correspond to a reduced model representing only half of the structure, whereas the numerical analysis was performed on the complete structure.

## 3. Discussion

The experimental results obtained from both the cantilever beam and the pedestrian footbridge confirm the viability of the proposed hybrid measurement system that combines Laser Doppler Vibrometry (LDV) with conventional accelerometers for operational modal analysis. The integration of velocity, acceleration, and displacement data enabled the consistent identification of modal parameters, thereby demonstrating the compatibility of the two sensor types.

One of the main advantages of the system is the non-contact capability of the LDV, which simplifies the instrumentation process in scenarios where direct access is restricted, or physical installation of sensors is impractical. In the footbridge test, reflective targets allowed sequential LDV measurements at multiple locations without the need to deploy numerous accelerometers, cables, and power supplies. Once installed, these targets can remain in place and be reused for repeated tests, providing significant efficiency for long-term monitoring campaigns. This feature makes the hybrid approach attractive not only for short-term measurements but also for periodic assessments of structural health.

The tests also highlighted certain limitations. Since uniaxial LDVs measure only along the direction of the laser beam, vertical vibrations had to be projected onto the horizontal axis by angling the targets. While effective in the laboratory, this procedure may become challenging in field applications, where alignment accuracy can be affected by geometry and environmental conditions. In medium to large structures, the integration of topographic or optical alignment tools could help to refine laser positioning and improve measurement precision. Although LDV measurements are acquired sequentially rather than simultaneously, which can extend the duration of a test campaign, the approach requires far fewer resources than dense accelerometer networks. A single LDV, combined with a reference accelerometer, can effectively replace numerous contact sensors, cabling, and acquisition channels, significantly simplifying the experimental setup.

Overall, the study demonstrates that the hybrid LDV–accelerometer system is not only technically feasible but also offers tangible benefits for modal analysis. It reduces the need for extensive sensor deployment, facilitates re-measurements through reusable targets, and provides reliable modal parameter estimation. At the same time, its limitations highlight opportunities for future refinement, particularly regarding alignment strategies and field deployment.

## 4. Conclusions

Based on the experimental program, the following conclusions can be drawn:

- The study demonstrates an innovative hybrid measurement strategy that combines LDV with a single reference accelerometer. This method was successfully validated on both a cantilever beam and a pedestrian laboratory footbridge, showing that reliable modal parameters can be obtained with a minimal number of contact sensors.
- The methodology enabled accurate identification of modal parameters, with minimal errors in natural frequencies (<2%) and reasonable agreement of mode shapes (MAC > 0.9). This confirms the engineering applicability of the proposed approach for OMA in civil structures.
- The integration of LDV and accelerometers ensured consistency across displacement, velocity, and acceleration domains, confirming the compatibility of the two systems and supporting their combined use in practical modal testing.
- The LDV’s non-contact capability, together with the use of reflective targets, simplified the experimental setup. The reusability of targets offers a practical advantage for repeated campaigns and long-term monitoring.
- The system is particularly suitable when direct sensor installation is impractical or when access to measurement points is restricted, and it is applicable to both vertical and horizontal vibration modes. This highlights its potential for complex or difficult-to-access civil engineering structures.
- Potential challenges include alignment of the laser beam, environmental influences, and the sequential nature of LDV data acquisition. Complementary topographic equipment may help overcome these issues in field applications.

Overall, the hybrid LDV–accelerometer system represents a flexible, scalable, and efficient solution for operational modal analysis and structural health monitoring of civil engineering structures. Potential future research directions include extending the methodology to full-scale bridges, evaluating its performance under ambient environmental conditions and automating LDV scanning paths.

## Figures and Tables

**Table 1:** Natural frequencies obtained only with accelerometers and with accelerometer + LDV.

Mode	Frequency [Hz]		Error [%]
	Accelerometer	Accelerometer + LDV	
1	2.686	2.653	1.22
2	17.460	17.697	1.36
3	49.190	48.667	1.06

**Table 2:** Natural frequencies obtained only with accelerometers and with accelerometers + LDV.

Mode	Frequency [Hz]		Error [%]
	Accelerometers	Accelerometer + LDV	
1	11.76	11.62	1.19
2	24.77	25.05	1.13
3	27.69	27.99	1.08
4	36.33	35.83	1.38

**Table 3:** Modal Assurance Criteria.

MAC			
0.9100	0.0026	0.0616	0.0018
0.0037	0.9500	0.0393	0.8082
0.0095	0.0365	0.9050	0.0314
0.0052	0.6468	0.0218	0.9450

**Table 4:** Experimental mode shapes compared with the numerical mode shapes from [38].

Mode	Experimental: LDV + Acc. Partial Half-Structure	Numerical Complete Structure
1 (Bending)	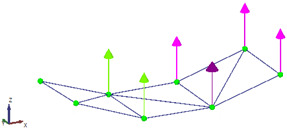	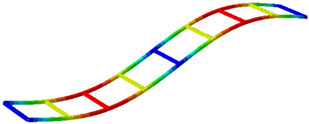
2 (Torsion)	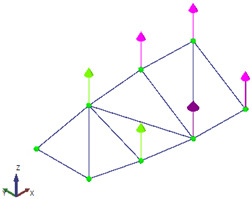	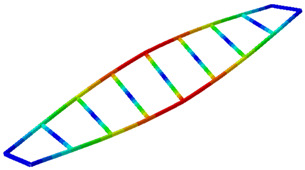
3 (Bending)	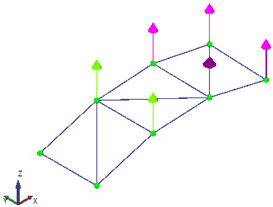	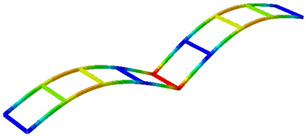
4 (Torsion)	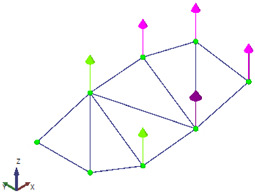	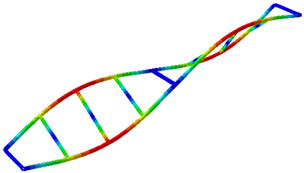

## Data Availability

Data will be made available on request.
